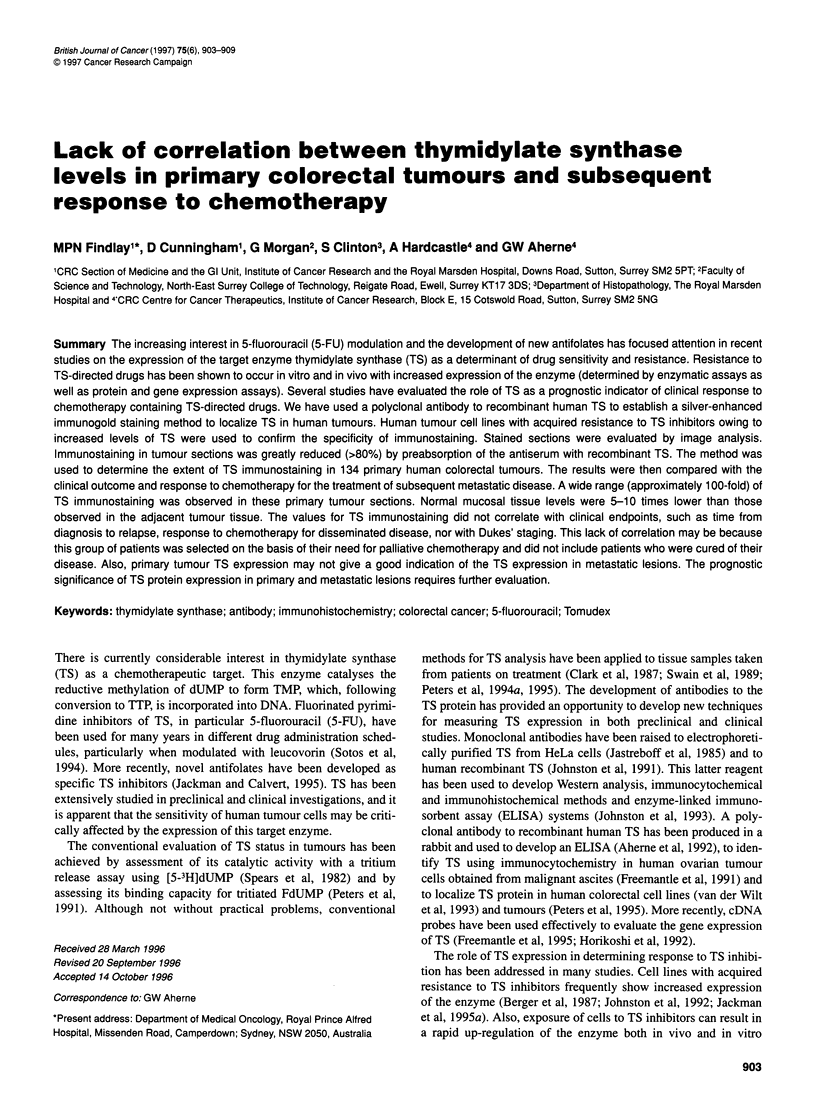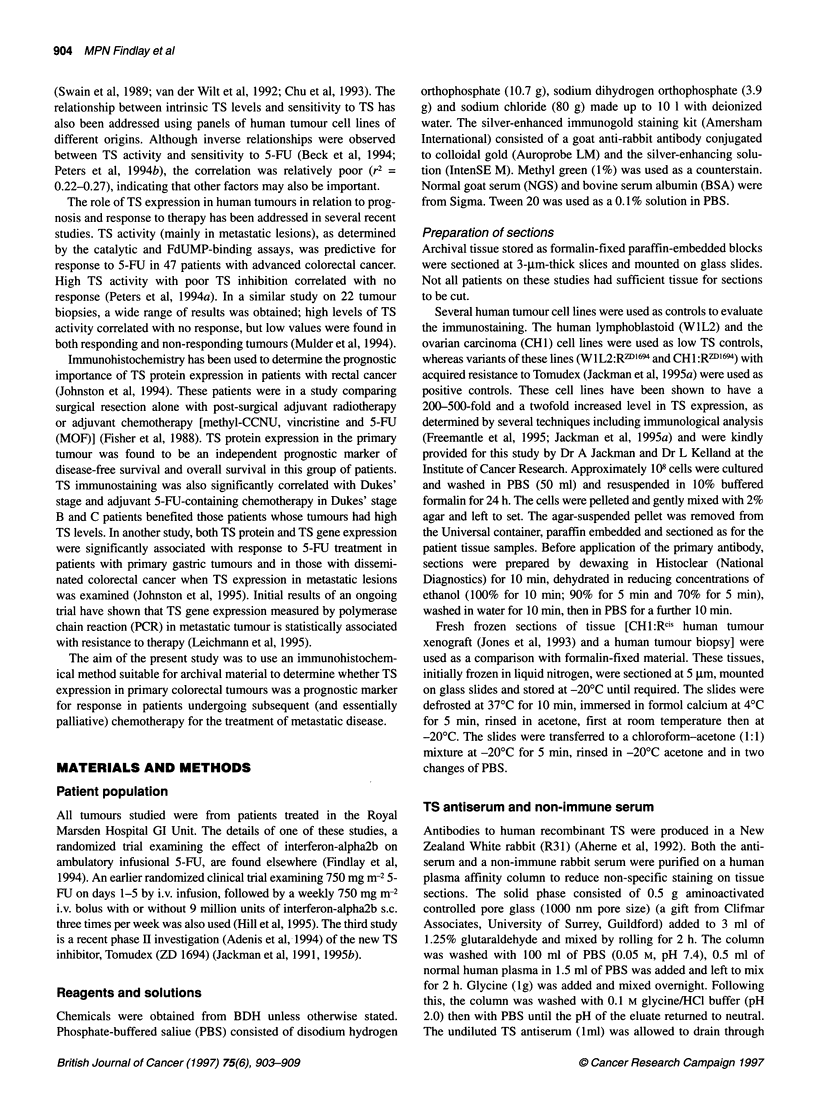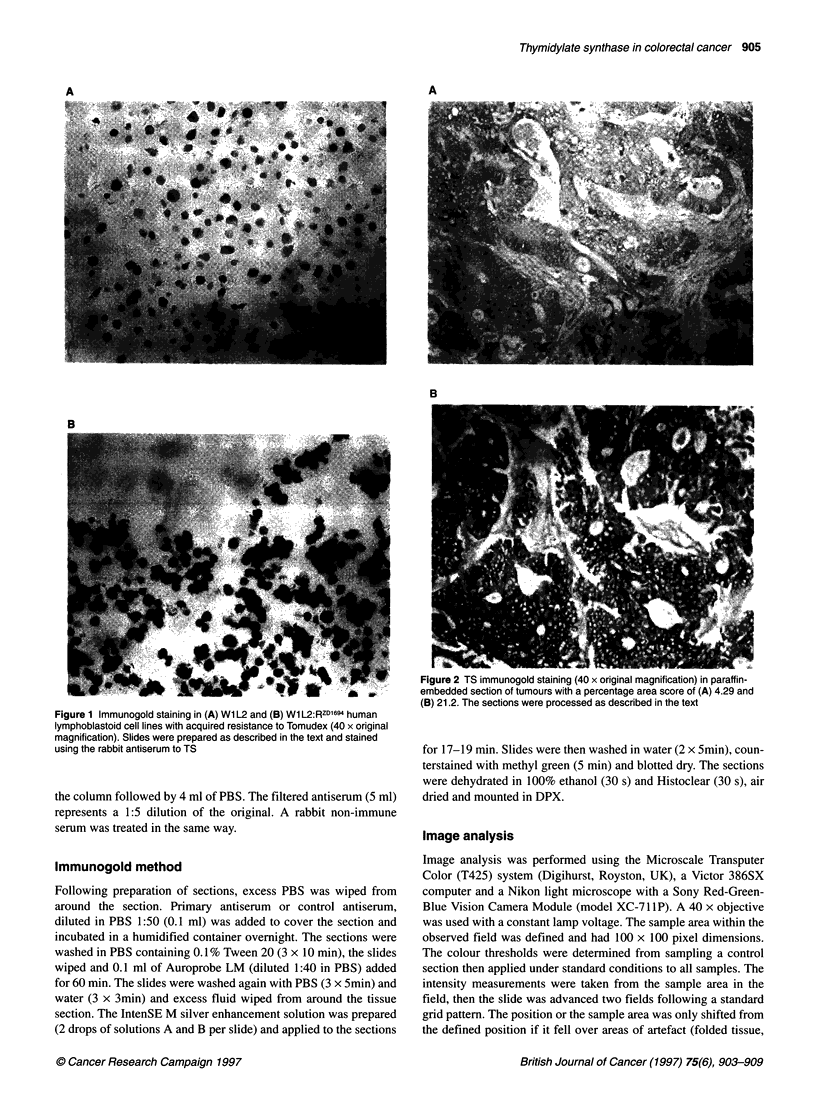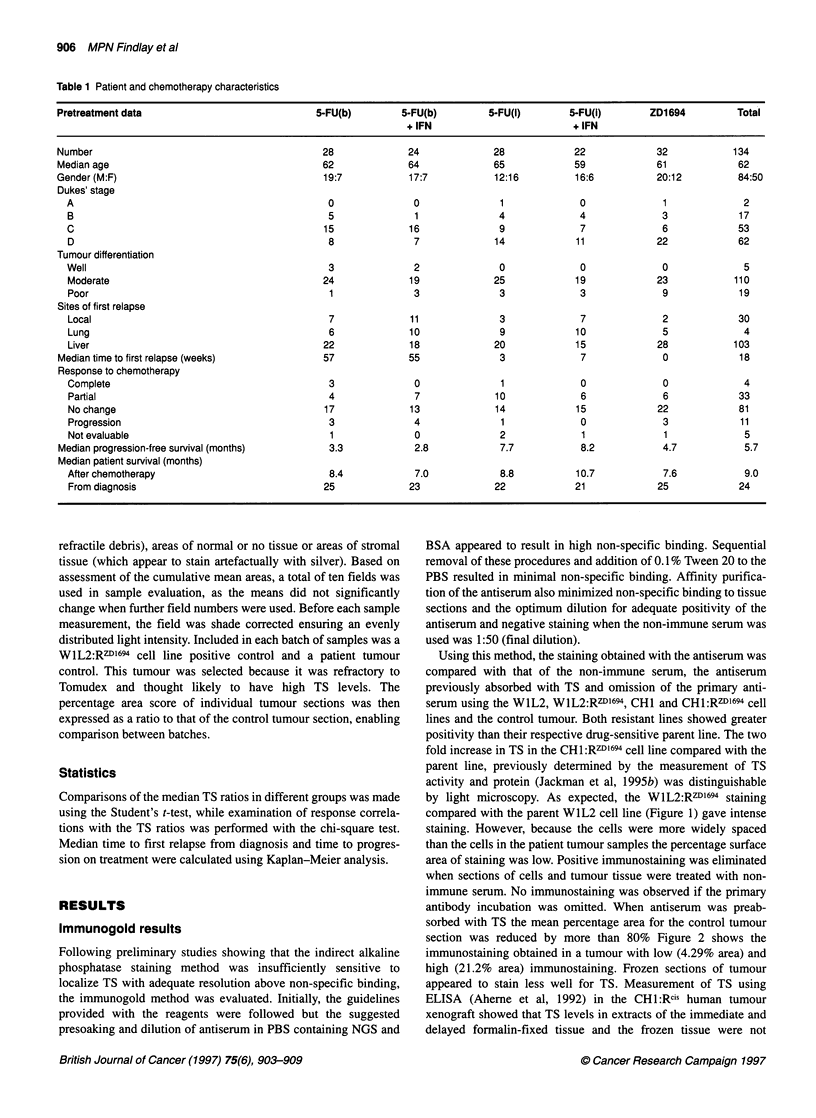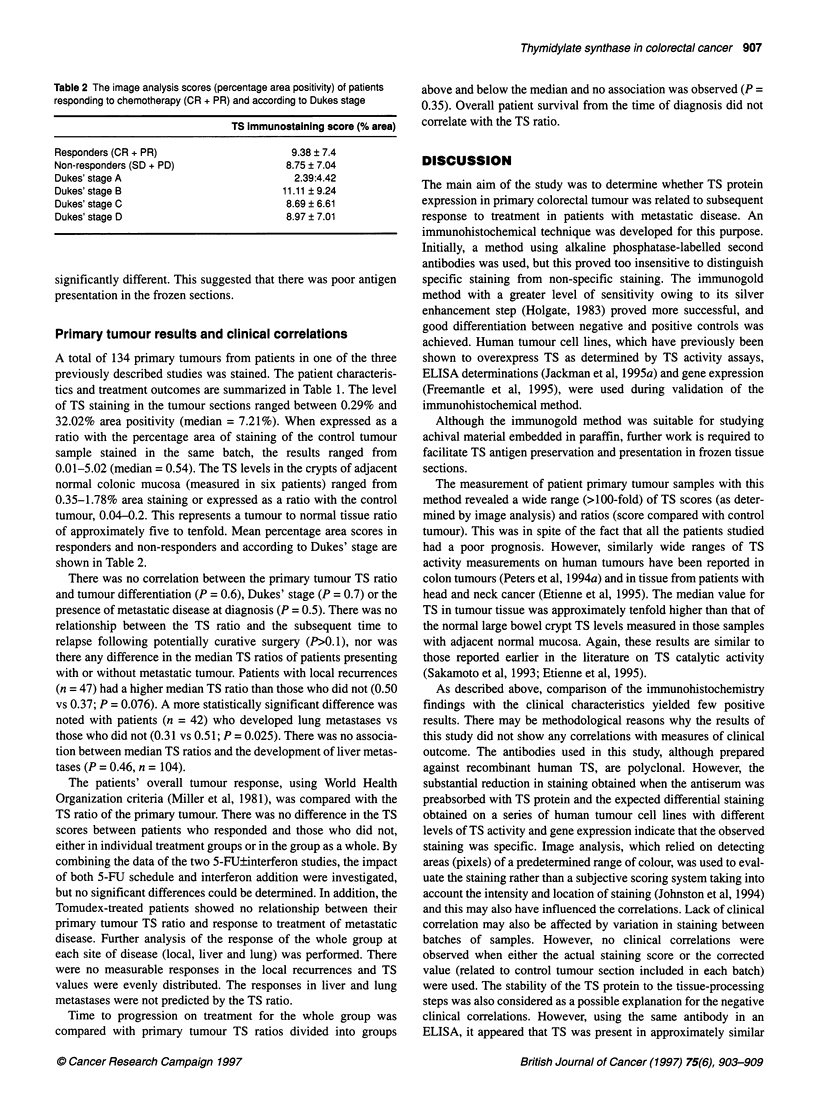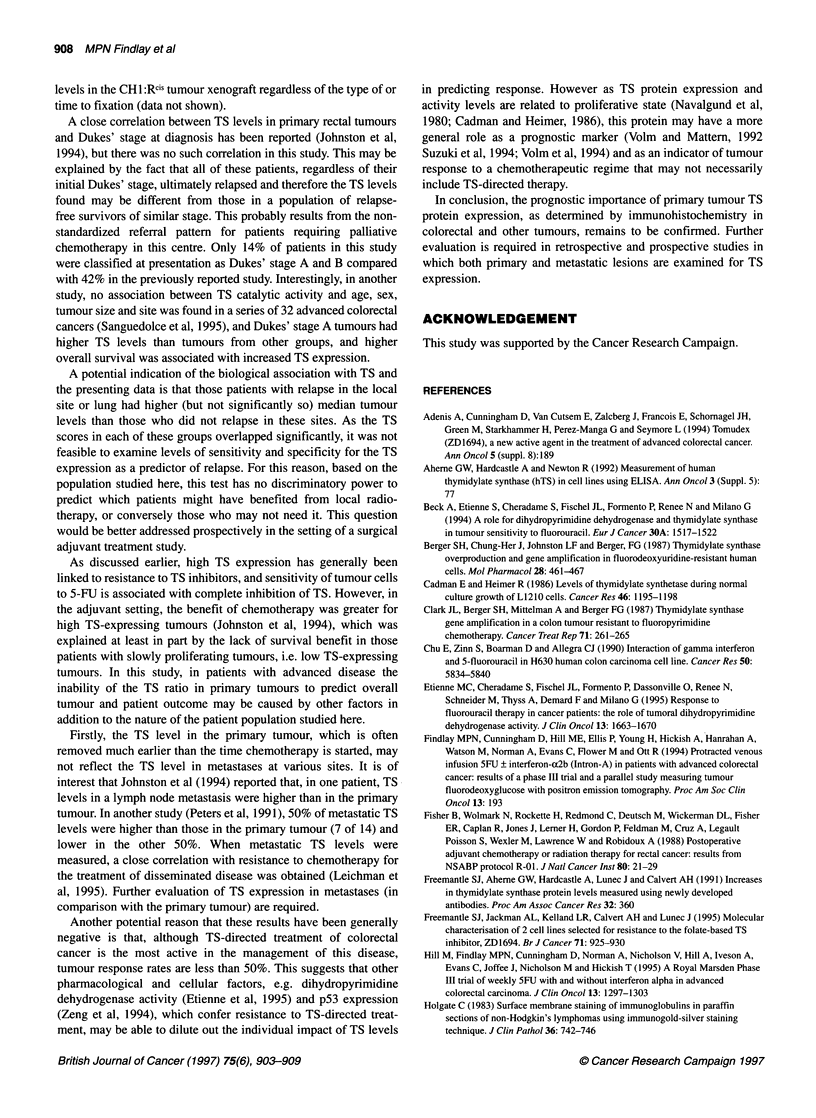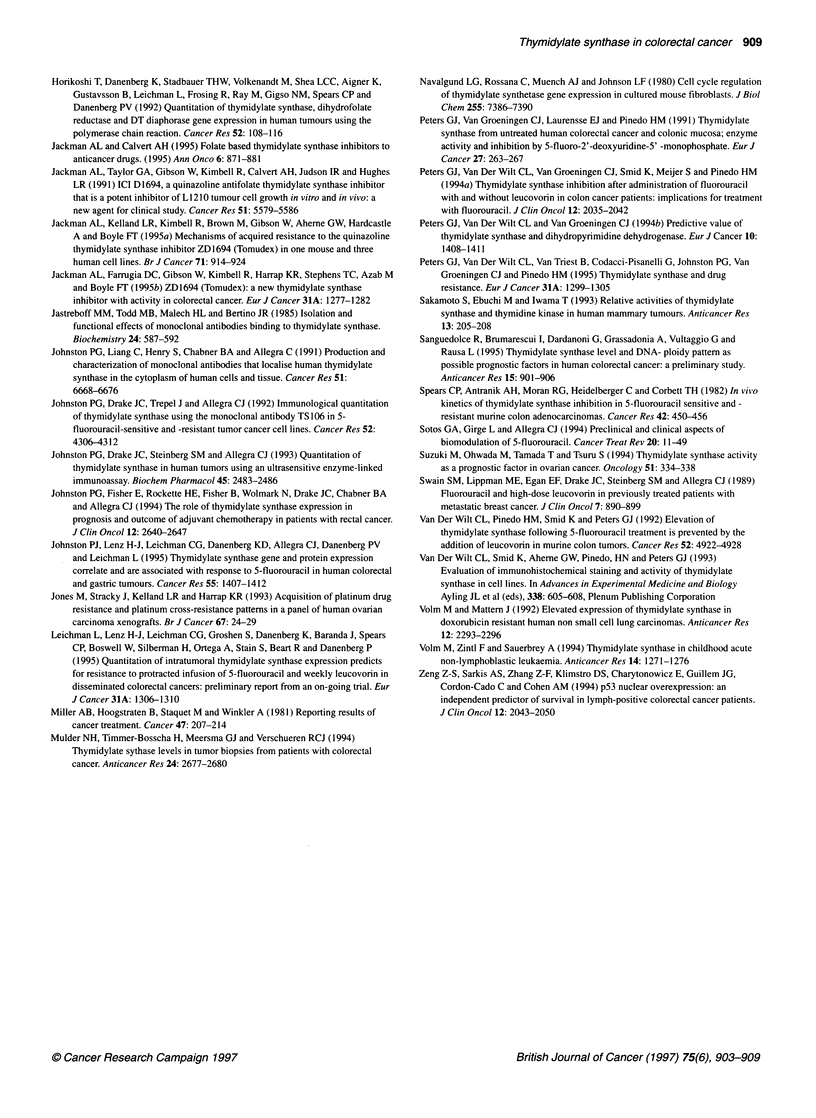# Lack of correlation between thymidylate synthase levels in primary colorectal tumours and subsequent response to chemotherapy.

**DOI:** 10.1038/bjc.1997.159

**Published:** 1997

**Authors:** M. P. Findlay, D. Cunningham, G. Morgan, S. Clinton, A. Hardcastle, G. W. Aherne

**Affiliations:** Institute of Cancer Research and the Royal Marsden Hospital, Sutton, Surrey.

## Abstract

**Images:**


British Journal of Cancer (1997) 75(6), 903–909
© 1997 Cancer Research Campaign

### Lack of correlation between thymidylate synthase levels in primary colorectal tumours and subsequent response to chemotherapy

**MPN Findlay[1*], D Cunningham[1], G Morgan[2], S Clinton[3], A Hardcastle[4] and GW Aherne[4]**

[1]CRC Section of Medicine and the GI Unit, Institute of Cancer Research and the Royal Marsden Hospital, Downs Road, Sutton, Surrey SM2 5PT; [2]Faculty of Science and Technology, North-East Surrey College of Technology, Reigate Road, Ewell, Surrey KT17 3DS; [3]Department of Histopathology, The Royal Marsden Hospital and [4]CRC Centre for Cancer Therapeutics, Institute of Cancer Research, Block E, 15 Cotswold Road, Sutton, Surrey SM2 5NG

**Summary** The increasing interest in 5-fluorouracil (5-FU) modulation and the development of new antifolates has focused attention in recent studies on the expression of the target enzyme thymidylate synthase (TS) as a determinant of drug sensitivity and resistance. Resistance to TS-directed drugs has been shown to occur in vitro and in vivo with increased expression of the enzyme (determined by enzymatic assays as well as protein and gene expression assays). Several studies have evaluated the role of TS as a prognostic indicator of clinical response to chemotherapy containing TS-directed drugs. We have used a polyclonal antibody to recombinant human TS to establish a silver-enhanced immunogold staining method to localize TS in human tumours. Human tumour cell lines with acquired resistance to TS inhibitors owing to increased levels of TS were used to confirm the specificity of immunostaining. Stained sections were evaluated by image analysis. Immunostaining in tumour sections was greatly reduced (>80%) by preabsorption of the antiserum with recombinant TS. The method was used to determine the extent of TS immunostaining in 134 primary human colorectal tumours. The results were then compared with the clinical outcome and response to chemotherapy for the treatment of subsequent metastatic disease. A wide range (approximately 100-fold) of TS immunostaining was observed in these primary tumour sections. Normal mucosal tissue levels were 5–10 times lower than those observed in the adjacent tumour tissue. The values for TS immunostaining did not correlate with clinical endpoints, such as time from diagnosis to relapse, response to chemotherapy for disseminated disease, nor with Dukes' staging. This lack of correlation may be because this group of patients was selected on the basis of their need for palliative chemotherapy and did not include patients who were cured of their disease. Also, primary tumour TS expression may not give a good indication of the TS expression in metastatic lesions. The prognostic significance of TS protein expression in primary and metastatic lesions requires further evaluation.

**Keywords:** thymidylate synthase; antibody; immunohistochemistry; colorectal cancer; 5-fluorouracil; Tomudex

There is currently considerable interest in thymidylate synthase (TS) as a chemotherapeutic target. This enzyme catalyses the reductive methylation of dUMP to form TMP, which, following conversion to TTP, is incorporated into DNA. Fluorinated pyrimidine inhibitors of TS, in particular 5-fluorouracil (5-FU), have been used for many years in different drug administration schedules, particularly when modulated with leucovorin (Sotos et al, 1994). More recently, novel antifolates have been developed as specific TS inhibitors (Jackman and Calvert, 1995). TS has been extensively studied in preclinical and clinical investigations, and it is apparent that the sensitivity of human tumour cells may be critically affected by the expression of this target enzyme.

The conventional evaluation of TS status in tumours has been achieved by assessment of its catalytic activity with a tritium release assay using [5-$^3$H]dUMP (Spears et al, 1982) and by assessing its binding capacity for tritiated FdUMP (Peters et al, 1991). Although not without practical problems, conventional methods for TS analysis have been applied to tissue samples taken from patients on treatment (Clark et al, 1987; Swain et al, 1989; Peters et al, 1994*a*, 1995). The development of antibodies to the TS protein has provided an opportunity to develop new techniques for measuring TS expression in both preclinical and clinical studies. Monoclonal antibodies have been raised to electrophoretically purified TS from HeLa cells (Jastreboff et al, 1985) and to human recombinant TS (Johnston et al, 1991). This latter reagent has been used to develop Western analysis, immunocytochemical and immunohistochemical methods and enzyme-linked immunosorbent assay (ELISA) systems (Johnston et al, 1993). A polyclonal antibody to recombinant human TS has been produced in a rabbit and used to develop an ELISA (Aherne et al, 1992), to identify TS using immunocytochemistry in human ovarian tumour cells obtained from malignant ascites (Freemantle et al, 1991) and to localize TS protein in human colorectal cell lines (van der Wilt et al, 1993) and tumours (Peters et al, 1995). More recently, cDNA probes have been used effectively to evaluate the gene expression of TS (Freemantle et al, 1995; Horikoshi et al, 1992).

The role of TS expression in determining response to TS inhibition has been addressed in many studies. Cell lines with acquired resistance to TS inhibitors frequently show increased expression of the enzyme (Berger et al, 1987; Johnston et al, 1992; Jackman et al, 1995*a*). Also, exposure of cells to TS inhibitors can result in a rapid up-regulation of the enzyme both in vivo and in vitro

*Received 28 March 1996*
*Revised 20 September 1996*
*Accepted 14 October 1996*

*Correspondence to:* GW Aherne

*Present address: Department of Medical Oncology, Royal Prince Alfred Hospital, Missenden Road, Camperdown; Sydney, NSW 2050, Australia

(Swain et al, 1989; van der Wilt et al, 1992; Chu et al, 1993). The relationship between intrinsic TS levels and sensitivity to TS has also been addressed using panels of human tumour cell lines of different origins. Although inverse relationships were observed between TS activity and sensitivity to 5-FU (Beck et al, 1994; Peters et al, 1994*b*), the correlation was relatively poor ($r^2$ = 0.22–0.27), indicating that other factors may also be important.

The role of TS expression in human tumours in relation to prognosis and response to therapy has been addressed in several recent studies. TS activity (mainly in metastatic lesions), as determined by the catalytic and FdUMP-binding assays, was predictive for response to 5-FU in 47 patients with advanced colorectal cancer. High TS activity with poor TS inhibition correlated with no response (Peters et al, 1994*a*). In a similar study on 22 tumour biopsies, a wide range of results was obtained; high levels of TS activity correlated with no response, but low values were found in both responding and non-responding tumours (Mulder et al, 1994).

Immunohistochemistry has been used to determine the prognostic importance of TS protein expression in patients with rectal cancer (Johnston et al, 1994). These patients were in a study comparing surgical resection alone with post-surgical adjuvant radiotherapy or adjuvant chemotherapy [methyl-CCNU, vincristine and 5-FU (MOF)] (Fisher et al, 1988). TS protein expression in the primary tumour was found to be an independent prognostic marker of disease-free survival and overall survival in this group of patients. TS immunostaining was also significantly correlated with Dukes' stage and adjuvant 5-FU-containing chemotherapy in Dukes' stage B and C patients benefited those patients whose tumours had high TS levels. In another study, both TS protein and TS gene expression were significantly associated with response to 5-FU treatment in patients with primary gastric tumours and in those with disseminated colorectal cancer when TS expression in metastatic lesions was examined (Johnston et al, 1995). Initial results of an ongoing trial have shown that TS gene expression measured by polymerase chain reaction (PCR) in metastatic tumour is statistically associated with resistance to therapy (Leichmann et al, 1995).

The aim of the present study was to use an immunohistochemical method suitable for archival material to determine whether TS expression in primary colorectal tumours was a prognostic marker for response in patients undergoing subsequent (and essentially palliative) chemotherapy for the treatment of metastatic disease.

### MATERIALS AND METHODS

### Patient population

All tumours studied were from patients treated in the Royal Marsden Hospital GI Unit. The details of one of these studies, a randomized trial examining the effect of interferon-alpha2b on ambulatory infusional 5-FU, are found elsewhere (Findlay et al, 1994). An earlier randomized clinical trial examining 750 mg $m^{-2}$ 5-FU on days 1–5 by i.v. infusion, followed by a weekly 750 mg $m^{-2}$ i.v. bolus with or without 9 million units of interferon-alpha2b s.c. three times per week was also used (Hill et al, 1995). The third study is a recent phase II investigation (Adenis et al, 1994) of the new TS inhibitor, Tomudex (ZD 1694) (Jackman et al, 1991, 1995*b*).

### Reagents and solutions

Chemicals were obtained from BDH unless otherwise stated. Phosphate-buffered saliue (PBS) consisted of disodium hydrogen orthophosphate (10.7 g), sodium dihydrogen orthophosphate (3.9 g) and sodium chloride (80 g) made up to 10 l with deionized water. The silver-enhanced immunogold staining kit (Amersham International) consisted of a goat anti-rabbit antibody conjugated to colloidal gold (Auroprobe LM) and the silver-enhancing solution (IntenSE M). Methyl green (1%) was used as a counterstain. Normal goat serum (NGS) and bovine serum albumin (BSA) were from Sigma. Tween 20 was used as a 0.1% solution in PBS.

### *Preparation of sections*

Archival tissue stored as formalin-fixed paraffin-embedded blocks were sectioned at 3-μm-thick slices and mounted on glass slides. Not all patients on these studies had sufficient tissue for sections to be cut.

Several human tumour cell lines were used as controls to evaluate the immunostaining. The human lymphoblastoid (W1L2) and the ovarian carcinoma (CH1) cell lines were used as low TS controls, whereas variants of these lines (W1L2:$R^{ZD1694}$ and CH1:$R^{ZD1694}$) with acquired resistance to Tomudex (Jackman et al, 1995*a*) were used as positive controls. These cell lines have been shown to have a 200–500-fold and a twofold increased level in TS expression, as determined by several techniques including immunological analysis (Freemantle et al, 1995; Jackman et al, 1995*a*) and were kindly provided for this study by Dr A Jackman and Dr L Kelland at the Institute of Cancer Research. Approximately $10^8$ cells were cultured and washed in PBS (50 ml) and resuspended in 10% buffered formalin for 24 h. The cells were pelleted and gently mixed with 2% agar and left to set. The agar-suspended pellet was removed from the Universal container, paraffin embedded and sectioned as for the patient tissue samples. Before application of the primary antibody, sections were prepared by dewaxing in Histoclear (National Diagnostics) for 10 min, dehydrated in reducing concentrations of ethanol (100% for 10 min; 90% for 5 min and 70% for 5 min), washed in water for 10 min, then in PBS for a further 10 min.

Fresh frozen sections of tissue [CH1:$R^{cis}$ human tumour xenograft (Jones et al, 1993) and a human tumour biopsy] were used as a comparison with formalin-fixed material. These tissues, initially frozen in liquid nitrogen, were sectioned at 5 μm, mounted on glass slides and stored at –20°C until required. The slides were defrosted at 37°C for 10 min, immersed in formol calcium at 4°C for 5 min, rinsed in acetone, first at room temperature then at –20°C. The slides were transferred to a chloroform–acetone (1:1) mixture at –20°C for 5 min, rinsed in –20°C acetone and in two changes of PBS.

### TS antiserum and non-immune serum

Antibodies to human recombinant TS were produced in a New Zealand White rabbit (R31) (Aherne et al, 1992). Both the antiserum and a non-immune rabbit serum were purified on a human plasma affinity column to reduce non-specific staining on tissue sections. The solid phase consisted of 0.5 g aminoactivated controlled pore glass (1000 nm pore size) (a gift from Clifmar Associates, University of Surrey, Guildford) added to 3 ml of 1.25% glutaraldehyde and mixed by rolling for 2 h. The column was washed with 100 ml of PBS (0.05 M, pH 7.4), 0.5 ml of normal human plasma in 1.5 ml of PBS was added and left to mix for 2 h. Glycine (1g) was added and mixed overnight. Following this, the column was washed with 0.1 M glycine/HCl buffer (pH 2.0) then with PBS until the pH of the eluate returned to neutral. The undiluted TS antiserum (1ml) was allowed to drain through

 © Cancer Research Campaign 1997

**Figure 1** Immunogold staining in (**A**) W1L2 and (**B**) W1L2:R$^{ZD1694}$ human lymphoblastoid cell lines with acquired resistance to Tomudex (40 × original magnification). Slides were prepared as described in the text and stained using the rabbit antiserum to TS

**Figure 2** TS immunogold staining (40 × original magnification) in paraffin-embedded section of tumours with a percentage area score of (**A**) 4.29 and (**B**) 21.2. The sections were processed as described in the text the column followed by 4 ml of PBS. The filtered antiserum (5 ml) represents a 1:5 dilution of the original. A rabbit non-immune serum was treated in the same way.

### Immunogold method

Following preparation of sections, excess PBS was wiped from around the section. Primary antiserum or control antiserum, diluted in PBS 1:50 (0.1 ml) was added to cover the section and incubated in a humidified container overnight. The sections were washed in PBS containing 0.1% Tween 20 (3 × 10 min), the slides wiped and 0.1 ml of Auroprobe LM (diluted 1:40 in PBS) added for 60 min. The slides were washed again with PBS (3 × 5min) and water (3 × 3min) and excess fluid wiped from around the tissue section. The IntenSE M silver enhancement solution was prepared (2 drops of solutions A and B per slide) and applied to the sections for 17–19 min. Slides were then washed in water (2 × 5min), counterstained with methyl green (5 min) and blotted dry. The sections were dehydrated in 100% ethanol (30 s) and Histoclear (30 s), air dried and mounted in DPX.

### Image analysis

Image analysis was performed using the Microscale Transputer Color (T425) system (Digihurst, Royston, UK), a Victor 386SX computer and a Nikon light microscope with a Sony Red-Green-Blue Vision Camera Module (model XC-711P). A 40 × objective was used with a constant lamp voltage. The sample area within the observed field was defined and had 100 × 100 pixel dimensions. The colour thresholds were determined from sampling a control section then applied under standard conditions to all samples. The intensity measurements were taken from the sample area in the field, then the slide was advanced two fields following a standard grid pattern. The position or the sample area was only shifted from the defined position if it fell over areas of artefact (folded tissue,

© Cancer Research Campaign 1997

**Table 1** Patient and chemotherapy characteristics

| Pretreatment data | 5-FU(b) | 5-FU(b) + IFN | 5-FU(i) | 5-FU(i) + IFN | ZD1694 | Total |
|---|---|---|---|---|---|---|
| Number | 28 | 24 | 28 | 22 | 32 | 134 |
| Median age | 62 | 64 | 65 | 59 | 61 | 62 |
| Gender (M:F) | 19:7 | 17:7 | 12:16 | 16:6 | 20:12 | 84:50 |
| Dukes' stage | | | | | | |
| A | 0 | 0 | 1 | 0 | 1 | 2 |
| B | 5 | 1 | 4 | 4 | 3 | 17 |
| C | 15 | 16 | 9 | 7 | 6 | 53 |
| D | 8 | 7 | 14 | 11 | 22 | 62 |
| Tumour differentiation | | | | | | |
| Well | 3 | 2 | 0 | 0 | 0 | 5 |
| Moderate | 24 | 19 | 25 | 19 | 23 | 110 |
| Poor | 1 | 3 | 3 | 3 | 9 | 19 |
| Sites of first relapse | | | | | | |
| Local | 7 | 11 | 3 | 7 | 2 | 30 |
| Lung | 6 | 10 | 9 | 10 | 5 | 4 |
| Liver | 22 | 18 | 20 | 15 | 28 | 103 |
| Median time to first relapse (weeks) | 57 | 55 | 3 | 7 | 0 | 18 |
| Response to chemotherapy | | | | | | |
| Complete | 3 | 0 | 1 | 0 | 0 | 4 |
| Partial | 4 | 7 | 10 | 6 | 6 | 33 |
| No change | 17 | 13 | 14 | 15 | 22 | 81 |
| Progression | 3 | 4 | 1 | 0 | 3 | 11 |
| Not evaluable | 1 | 0 | 2 | 1 | 1 | 5 |
| Median progression-free survival (months) | 3.3 | 2.8 | 7.7 | 8.2 | 4.7 | 5.7 |
| Median patient survival (months) | | | | | | |
| After chemotherapy | 8.4 | 7.0 | 8.8 | 10.7 | 7.6 | 9.0 |
| From diagnosis | 25 | 23 | 22 | 21 | 25 | 24 |

refractile debris), areas of normal or no tissue or areas of stromal tissue (which appear to stain artefactually with silver). Based on assessment of the cumulative mean areas, a total of ten fields was used in sample evaluation, as the means did not significantly change when further field numbers were used. Before each sample measurement, the field was shade corrected ensuring an evenly distributed light intensity. Included in each batch of samples was a W1L2:$R^{ZD1694}$ cell line positive control and a patient tumour control. This tumour was selected because it was refractory to Tomudex and thought likely to have high TS levels. The percentage area score of individual tumour sections was then expressed as a ratio to that of the control tumour section, enabling comparison between batches.

### Statistics

Comparisons of the median TS ratios in different groups was made using the Student's *t*-test, while examination of response correlations with the TS ratios was performed with the chi-square test. Median time to first relapse from diagnosis and time to progression on treatment were calculated using Kaplan–Meier analysis.

### RESULTS

### Immunogold results

Following preliminary studies showing that the indirect alkaline phosphatase staining method was insufficiently sensitive to localize TS with adequate resolution above non-specific binding, the immunogold method was evaluated. Initially, the guidelines provided with the reagents were followed but the suggested presoaking and dilution of antiserum in PBS containing NGS and BSA appeared to result in high non-specific binding. Sequential removal of these procedures and addition of 0.1% Tween 20 to the PBS resulted in minimal non-specific binding. Affinity purification of the antiserum also minimized non-specific binding to tissue sections and the optimum dilution for adequate positivity of the antiserum and negative staining when the non-immune serum was used was 1:50 (final dilution).

Using this method, the staining obtained with the antiserum was compared with that of the non-immune serum, the antiserum previously absorbed with TS and omission of the primary antiserum using the W1L2, W1L2:$R^{ZD1694}$, CH1 and CH1:$R^{ZD1694}$ cell lines and the control tumour. Both resistant lines showed greater positivity than their respective drug-sensitive parent line. The two fold increase in TS in the CH1:$R^{ZD1694}$ cell line compared with the parent line, previously determined by the measurement of TS activity and protein (Jackman et al, 1995*b*) was distinguishable by light microscopy. As expected, the W1L2:$R^{ZD1694}$ staining compared with the parent W1L2 cell line (Figure 1) gave intense staining. However, because the cells were more widely spaced than the cells in the patient tumour samples the percentage surface area of staining was low. Positive immunostaining was eliminated when sections of cells and tumour tissue were treated with non-immune serum. No immunostaining was observed if the primary antibody incubation was omitted. When antiserum was preabsorbed with TS the mean percentage area for the control tumour section was reduced by more than 80% Figure 2 shows the immunostaining obtained in a tumour with low (4.29% area) and high (21.2% area) immunostaining. Frozen sections of tumour appeared to stain less well for TS. Measurement of TS using ELISA (Aherne et al, 1992) in the CH1:$R^{cis}$ human tumour xenograft showed that TS levels in extracts of the immediate and delayed formalin-fixed tissue and the frozen tissue were not

 © Cancer Research Campaign 1997

Table 2 The image analysis scores (percentage area positivity) of patients responding to chemotherapy (CR + PR) and according to Dukes stage

| | TS immunostaining score (% area) |
|---|---|
| Responders (CR + PR) | 9.38 ± 7.4 |
| Non-responders (SD + PD) | 8.75 ± 7.04 |
| Dukes' stage A | 2.39:4.42 |
| Dukes' stage B | 11.11 ± 9.24 |
| Dukes' stage C | 8.69 ± 6.61 |
| Dukes' stage D | 8.97 ± 7.01 |

significantly different. This suggested that there was poor antigen presentation in the frozen sections.

### Primary tumour results and clinical correlations

A total of 134 primary tumours from patients in one of the three previously described studies was stained. The patient characteristics and treatment outcomes are summarized in Table 1. The level of TS staining in the tumour sections ranged between 0.29% and 32.02% area positivity (median = 7.21%). When expressed as a ratio with the percentage area of staining of the control tumour sample stained in the same batch, the results ranged from 0.01–5.02 (median = 0.54). The TS levels in the crypts of adjacent normal colonic mucosa (measured in six patients) ranged from 0.35–1.78% area staining or expressed as a ratio with the control tumour, 0.04–0.2. This represents a tumour to normal tissue ratio of approximately five to tenfold. Mean percentage area scores in responders and non-responders and according to Dukes' stage are shown in Table 2.

There was no correlation between the primary tumour TS ratio and tumour differentiation ($P = 0.6$), Dukes' stage ($P = 0.7$) or the presence of metastatic disease at diagnosis ($P = 0.5$). There was no relationship between the TS ratio and the subsequent time to relapse following potentially curative surgery ($P>0.1$), nor was there any difference in the median TS ratios of patients presenting with or without metastatic tumour. Patients with local recurrences ($n = 47$) had a higher median TS ratio than those who did not (0.50 vs 0.37; $P = 0.076$). A more statistically significant difference was noted with patients ($n = 42$) who developed lung metastases vs those who did not (0.31 vs 0.51; $P = 0.025$). There was no association between median TS ratios and the development of liver metastases ($P = 0.46$, $n = 104$).

The patients' overall tumour response, using World Health Organization criteria (Miller et al, 1981), was compared with the TS ratio of the primary tumour. There was no difference in the TS scores between patients who responded and those who did not, either in individual treatment groups or in the group as a whole. By combining the data of the two 5-FU±interferon studies, the impact of both 5-FU schedule and interferon addition were investigated, but no significant differences could be determined. In addition, the Tomudex-treated patients showed no relationship between their primary tumour TS ratio and response to treatment of metastatic disease. Further analysis of the response of the whole group at each site of disease (local, liver and lung) was performed. There were no measurable responses in the local recurrences and TS values were evenly distributed. The responses in liver and lung metastases were not predicted by the TS ratio.

Time to progression on treatment for the whole group was compared with primary tumour TS ratios divided into groups above and below the median and no association was observed ($P = 0.35$). Overall patient survival from the time of diagnosis did not correlate with the TS ratio.

### DISCUSSION

The main aim of the study was to determine whether TS protein expression in primary colorectal tumour was related to subsequent response to treatment in patients with metastatic disease. An immunohistochemical technique was developed for this purpose. Initially, a method using alkaline phosphatase-labelled second antibodies was used, but this proved too insensitive to distinguish specific staining from non-specific staining. The immunogold method with a greater level of sensitivity owing to its silver enhancement step (Holgate, 1983) proved more successful, and good differentiation between negative and positive controls was achieved. Human tumour cell lines, which have previously been shown to overexpress TS as determined by TS activity assays, ELISA determinations (Jackman et al, 1995*a*) and gene expression (Freemantle et al, 1995), were used during validation of the immunohistochemical method.

Although the immunogold method was suitable for studying achival material embedded in paraffin, further work is required to facilitate TS antigen preservation and presentation in frozen tissue sections.

The measurement of patient primary tumour samples with this method revealed a wide range (>100-fold) of TS scores (as determined by image analysis) and ratios (score compared with control tumour). This was in spite of the fact that all the patients studied had a poor prognosis. However, similarly wide ranges of TS activity measurements on human tumours have been reported in colon tumours (Peters et al, 1994*a*) and in tissue from patients with head and neck cancer (Etienne et al, 1995). The median value for TS in tumour tissue was approximately tenfold higher than that of the normal large bowel crypt TS levels measured in those samples with adjacent normal mucosa. Again, these results are similar to those reported earlier in the literature on TS catalytic activity (Sakamoto et al, 1993; Etienne et al, 1995).

As described above, comparison of the immunohistochemistry findings with the clinical characteristics yielded few positive results. There may be methodological reasons why the results of this study did not show any correlations with measures of clinical outcome. The antibodies used in this study, although prepared against recombinant human TS, are polyclonal. However, the substantial reduction in staining obtained when the antiserum was preabsorbed with TS protein and the expected differential staining obtained on a series of human tumour cell lines with different levels of TS activity and gene expression indicate that the observed staining was specific. Image analysis, which relied on detecting areas (pixels) of a predetermined range of colour, was used to evaluate the staining rather than a subjective scoring system taking into account the intensity and location of staining (Johnston et al, 1994) and this may also have influenced the correlations. Lack of clinical correlation may also be affected by variation in staining between batches of samples. However, no clinical correlations were observed when either the actual staining score or the corrected value (related to control tumour section included in each batch) were used. The stability of the TS protein to the tissue-processing steps was also considered as a possible explanation for the negative clinical correlations. However, using the same antibody in an ELISA, it appeared that TS was present in approximately similar

© Cancer Research Campaign 1997

levels in the CH1:R$^{cis}$ tumour xenograft regardless of the type of or time to fixation (data not shown).

A close correlation between TS levels in primary rectal tumours and Dukes' stage at diagnosis has been reported (Johnston et al, 1994), but there was no such correlation in this study. This may be explained by the fact that all of these patients, regardless of their initial Dukes' stage, ultimately relapsed and therefore the TS levels found may be different from those in a population of relapse-free survivors of similar stage. This probably results from the non-standardized referral pattern for patients requiring palliative chemotherapy in this centre. Only 14% of patients in this study were classified at presentation as Dukes' stage A and B compared with 42% in the previously reported study. Interestingly, in another study, no association between TS catalytic activity and age, sex, tumour size and site was found in a series of 32 advanced colorectal cancers (Sanguedolce et al, 1995), and Dukes' stage A tumours had higher TS levels than tumours from other groups, and higher overall survival was associated with increased TS expression.

A potential indication of the biological association with TS and the presenting data is that those patients with relapse in the local site or lung had higher (but not significantly so) median tumour levels than those who did not relapse in these sites. As the TS scores in each of these groups overlapped significantly, it was not feasible to examine levels of sensitivity and specificity for the TS expression as a predictor of relapse. For this reason, based on the population studied here, this test has no discriminatory power to predict which patients might have benefited from local radiotherapy, or conversely those who may not need it. This question would be better addressed prospectively in the setting of a surgical adjuvant treatment study.

As discussed earlier, high TS expression has generally been linked to resistance to TS inhibitors, and sensitivity of tumour cells to 5-FU is associated with complete inhibition of TS. However, in the adjuvant setting, the benefit of chemotherapy was greater for high TS-expressing tumours (Johnston et al, 1994), which was explained at least in part by the lack of survival benefit in those patients with slowly proliferating tumours, i.e. low TS-expressing tumours. In this study, in patients with advanced disease the inability of the TS ratio in primary tumours to predict overall tumour and patient outcome may be caused by other factors in addition to the nature of the patient population studied here.

Firstly, the TS level in the primary tumour, which is often removed much earlier than the time chemotherapy is started, may not reflect the TS level in metastases at various sites. It is of interest that Johnston et al (1994) reported that, in one patient, TS levels in a lymph node metastasis were higher than in the primary tumour. In another study (Peters et al, 1991), 50% of metastatic TS levels were higher than those in the primary tumour (7 of 14) and lower in the other 50%. When metastatic TS levels were measured, a close correlation with resistance to chemotherapy for the treatment of disseminated disease was obtained (Leichman et al, 1995). Further evaluation of TS expression in metastases (in comparison with the primary tumour) are required.

Another potential reason that these results have been generally negative is that, although TS-directed treatment of colorectal cancer is the most active in the management of this disease, tumour response rates are less than 50%. This suggests that other pharmacological and cellular factors, e.g. dihydropyrimidine dehydrogenase activity (Etienne et al, 1995) and p53 expression (Zeng et al, 1994), which confer resistance to TS-directed treatment, may be able to dilute out the individual impact of TS levels in predicting response. However as TS protein expression and activity levels are related to proliferative state (Navalgund et al, 1980; Cadman and Heimer, 1986), this protein may have a more general role as a prognostic marker (Volm and Mattern, 1992 Suzuki et al, 1994; Volm et al, 1994) and as an indicator of tumour response to a chemotherapeutic regime that may not necessarily include TS-directed therapy.

In conclusion, the prognostic importance of primary tumour TS protein expression, as determined by immunohistochemistry in colorectal and other tumours, remains to be confirmed. Further evaluation is required in retrospective and prospective studies in which both primary and metastatic lesions are examined for TS expression.

### ACKNOWLEDGEMENT

This study was supported by the Cancer Research Campaign.

### REFERENCES

Adenis A, Cunningham D, Van Cutsem E, Zalcberg J, Francois E, Schornagel JH, Green M, Starkhammer H, Perez-Manga G and Seymore L (1994) Tomudex (ZD1694), a new active agent in the treatment of advanced colorectal cancer. *Ann Oncol* **5** (suppl. 8):189

Aherne GW, Hardcastle A and Newton R (1992) Measurement of human thymidylate synthase (hTS) in cell lines using ELISA. *Ann Oncol* **3** (Suppl. 5): 77

Beck A, Etienne S, Cheradame S, Fischel JL, Formento P, Renee N and Milano G (1994) A role for dihydropyrimidine dehydrogenase and thymidylate synthase in tumour sensitivity to fluorouracil. *Eur J Cancer* **30A**: 1517–1522

Berger SH, Chung-Her J, Johnston LF and Berger, FG (1987) Thymidylate synthase overproduction and gene amplification in fluorodeoxyuridine-resistant human cells. *Mol Pharmacol* **28**: 461–467

Cadman E and Heimer R (1986) Levels of thymidylate synthetase during normal culture growth of L1210 cells. *Cancer Res* **46**: 1195–1198

Clark JL, Berger SH, Mittelman A and Berger FG (1987) Thymidylate synthase gene amplification in a colon tumour resistant to fluoropyrimidine chemotherapy. *Cancer Treat Rep* **71**: 261–265

Chu E, Zinn S, Boarman D and Allegra CJ (1990) Interaction of gamma interferon and 5-fluorouracil in H630 human colon carcinoma cell line. *Cancer Res* **50**: 5834–5840

Etienne MC, Cheradame S, Fischel JL, Formento P, Dassonville O, Renee N, Schneider M, Thyss A, Demard F and Milano G (1995) Response to fluorouracil therapy in cancer patients: the role of tumoral dihydropyrimidine dehydrogenase activity. *J Clin Oncol* **13**: 1663–1670

Findlay MPN, Cunningham D, Hill ME, Ellis P, Young H, Hickish A, Hanrahan A, Watson M, Norman A, Evans C, Flower M and Ott R (1994) Protracted venous infusion 5FU ± interferon-α2b (Intron-A) in patients with advanced colorectal cancer: results of a phase III trial and a parallel study measuring tumour fluorodeoxyglucose with positron emission tomography. *Proc Am Soc Clin Oncol* **13**: 193

Fisher B, Wolmark N, Rockette H, Redmond C, Deutsch M, Wickerman DL, Fisher ER, Caplan R, Jones J, Lerner H, Gordon P, Feldman M, Cruz A, Legault Poisson S, Wexler M, Lawrence W and Robidoux A (1988) Postoperative adjuvant chemotherapy or radiation therapy for rectal cancer: results from NSABP protocol R-01. *J Natl Cancer Inst* **80**: 21–29

Freemantle SJ, Aherne GW, Hardcastle A, Lunec J and Calvert AH (1991) Increases in thymidylate synthase protein levels measured using newly developed antibodies. *Proc Am Assoc Cancer Res* **32**: 360

Freemantle SJ, Jackman AL, Kelland LR, Calvert AH and Lunec J (1995) Molecular characterisation of 2 cell lines selected for resistance to the folate-based TS inhibitor, ZD1694. *Br J Cancer* **71**: 925–930

Hill M, Findlay MPN, Cunningham D, Norman A, Nicholson V, Hill A, Iveson A, Evans C, Joffee J, Nicholson M and Hickish T (1995) A Royal Marsden Phase III trial of weekly 5FU with and without interferon alpha in advanced colorectal carcinoma. *J Clin Oncol* **13**: 1297–1303

Holgate C (1983) Surface membrane staining of immunoglobulins in paraffin sections of non-Hodgkin's lymphomas using immunogold-silver staining technique. *J Clin Pathol* **36**: 742–746

© Cancer Research Campaign 1997

Horikoshi T, Danenberg K, Stadbauer THW, Volkenandt M, Shea LCC, Aigner K, Gustavsson B, Leichman L, Frosing R, Ray M, Gigso NM, Spears CP and Danenberg PV (1992) Quantitation of thymidylate synthase, dihydrofolate reductase and DT diaphorase gene expression in human tumours using the polymerase chain reaction. *Cancer Res* **52**: 108–116

Jackman AL and Calvert AH (1995) Folate based thymidylate synthase inhibitors to anticancer drugs. (1995) *Ann Onco* **6**: 871–881

Jackman AL, Taylor GA, Gibson W, Kimbell R, Calvert AH, Judson IR and Hughes LR (1991) ICI D1694, a quinazoline antifolate thymidylate synthase inhibitor that is a potent inhibitor of L1210 tumour cell growth *in vitro* and *in vivo*: a new agent for clinical study. *Cancer Res* **51**: 5579–5586

Jackman AL, Kelland LR, Kimbell R, Brown M, Gibson W, Aherne GW, Hardcastle A and Boyle FT (1995*a*) Mechanisms of acquired resistance to the quinazoline thymidylate synthase inhibitor ZD1694 (Tomudex) in one mouse and three human cell lines. *Br J Cancer* **71**: 914–924

Jackman AL, Farrugia DC, Gibson W, Kimbell R, Harrap KR, Stephens TC, Azab M and Boyle FT (1995*b*) ZD1694 (Tomudex): a new thymidylate synthase inhibitor with activity in colorectal cancer. *Eur J Cancer* **31A**: 1277–1282

Jastreboff MM, Todd MB, Malech HL and Bertino JR (1985) Isolation and functional effects of monoclonal antibodies binding to thymidylate synthase. *Biochemistry* **24**: 587–592

Johnston PG, Liang C, Henry S, Chabner BA and Allegra C (1991) Production and characterization of monoclonal antibodies that localise human thymidylate synthase in the cytoplasm of human cells and tissue. *Cancer Res* **51**: 6668–6676

Johnston PG, Drake JC, Trepel J and Allegra CJ (1992) Immunological quantitation of thymidylate synthase using the monoclonal antibody TS106 in 5-fluorouracil-sensitive and -resistant tumor cancer cell lines. *Cancer Res* **52**: 4306–4312

Johnston PG, Drake JC, Steinberg SM and Allegra CJ (1993) Quantitation of thymidylate synthase in human tumors using an ultrasensitive enzyme-linked immunoassay. *Biochem Pharmacol* **45**: 2483–2486

Johnston PG, Fisher E, Rockette HE, Fisher B, Wolmark N, Drake JC, Chabner BA and Allegra CJ (1994) The role of thymidylate synthase expression in prognosis and outcome of adjuvant chemotherapy in patients with rectal cancer. *J Clin Oncol* **12**: 2640–2647

Johnston PJ, Lenz H-J, Leichman CG, Danenberg KD, Allegra CJ, Danenberg PV and Leichman L (1995) Thymidylate synthase gene and protein expression correlate and are associated with response to 5-fluorouracil in human colorectal and gastric tumours. *Cancer Res* **55**: 1407–1412

Jones M, Stracky J, Kelland LR and Harrap KR (1993) Acquisition of platinum drug resistance and platinum cross-resistance patterns in a panel of human ovarian carcinoma xenografts. *Br J Cancer* **67**: 24–29

Leichman L, Lenz H-J, Leichman CG, Groshen S, Danenberg K, Baranda J, Spears CP, Boswell W, Silberman H, Ortega A, Stain S, Beart R and Danenberg P (1995) Quantitation of intratumoral thymidylate synthase expression predicts for resistance to protracted infusion of 5-fluorouracil and weekly leucovorin in disseminated colorectal cancers: preliminary report from an on-going trial. *Eur J Cancer* **31A**: 1306–1310

Miller AB, Hoogstraten B, Staquet M and Winkler A (1981) Reporting results of cancer treatment. *Cancer* **47**: 207–214

Mulder NH, Timmer-Bosscha H, Meersma GJ and Verschueren RCJ (1994) Thymidylate sythase levels in tumor biopsies from patients with colorectal cancer. *Anticancer Res* **24**: 2677–2680

Navalgund LG, Rossana C, Muench AJ and Johnson LF (1980) Cell cycle regulation of thymidylate synthetase gene expression in cultured mouse fibroblasts. *J Biol Chem* **255**: 7386–7390

Peters GJ, Van Groeningen CJ, Laurensse EJ and Pinedo HM (1991) Thymidylate synthase from untreated human colorectal cancer and colonic mucosa; enzyme activity and inhibition by 5-fluoro-2'-deoxyuridine-5' -monophosphate. *Eur J Cancer* **27**: 263–267

Peters GJ, Van Der Wilt CL, Van Groeningen CJ, Smid K, Meijer S and Pinedo HM (1994*a*) Thymidylate synthase inhibition after administration of fluorouracil with and without leucovorin in colon cancer patients: implications for treatment with fluorouracil. *J Clin Oncol* **12**: 2035–2042

Peters GJ, Van Der Wilt CL and Van Groeningen CJ (1994*b*) Predictive value of thymidylate synthase and dihydropyrimidine dehydrogenase. *Eur J* Cancer **10**: 1408–1411

Peters GJ, Van Der Wilt CL, Van Triest B, Codacci-Pisanelli G, Johnston PG, Van Groeningen CJ and Pinedo HM (1995) Thymidylate synthase and drug resistance. *Eur J Cancer* **31A**: 1299–1305

Sakamoto S, Ebuchi M and Iwama T (1993) Relative activities of thymidylate synthase and thymidine kinase in human mammary tumours. *Anticancer Res* **13**: 205–208

Sanguedolce R, Brumarescui I, Dardanoni G, Grassadonia A, Vultaggio G and Rausa L (1995) Thymidylate synthase level and DNA- ploidy pattern as possible prognostic factors in human colorectal cancer: a preliminary study. *Anticancer Res* **15**: 901–906

Spears CP, Antranik AH, Moran RG, Heidelberger C and Corbett TH (1982) *In vivo* kinetics of thymidylate synthase inhibition in 5-fluorouracil sensitive and -resistant murine colon adenocarcinomas. *Cancer Res* **42**: 450–456

Sotos GA, Girge L and Allegra CJ (1994) Preclinical and clinical aspects of biomodulation of 5-fluorouracil. *Cancer Treat Rev* **20**: 11–49

Suzuki M, Ohwada M, Tamada T and Tsuru S (1994) Thymidylate synthase activity as a prognostic factor in ovarian cancer. *Oncology* **51**: 334–338

Swain SM, Lippman ME, Egan EF, Drake JC, Steinberg SM and Allegra CJ (1989) Fluorouracil and high-dose leucovorin in previously treated patients with metastatic breast cancer. *J Clin Oncol* **7**: 890–899

Van Der Wilt CL, Pinedo HM, Smid K and Peters GJ (1992) Elevation of thymidylate synthase following 5-fluorouracil treatment is prevented by the addition of leucovorin in murine colon tumors. *Cancer Res* **52**: 4922–4928

Van Der Wilt CL, Smid K, Aherne GW, Pinedo, HN and Peters GJ (1993) Evaluation of immunohistochemical staining and activity of thymidylate synthase in cell lines. In *Advances in Experimental Medicine and Biology* Ayling JL et al (eds), **338**: 605–608, Plenum Publishing Corporation

Volm M and Mattern J (1992) Elevated expression of thymidylate synthase in doxorubicin resistant human non small cell lung carcinomas. *Anticancer Res* **12**: 2293–2296

Volm M, Zintl F and Sauerbrey A (1994) Thymidylate synthase in childhood acute non-lymphoblastic leukaemia. *Anticancer Res* **14**: 1271–1276

Zeng Z-S, Sarkis AS, Zhang Z-F, Klimstro DS, Charytonowicz E, Guillem JG, Cordon-Cado C and Cohen AM (1994) p53 nuclear overexpression: an independent predictor of survival in lymph-positive colorectal cancer patients. *J Clin Oncol* **12**: 2043–2050

© Cancer Research Campaign 1997